# Obstacle-Aware Crowd Surveillance with Mobile Robots in Transportation Stations

**DOI:** 10.3390/s25020350

**Published:** 2025-01-09

**Authors:** Yumin Choi, Hyunbum Kim

**Affiliations:** Department of Embedded Systems Engineering, Incheon National University, Incheon 22012, Republic of Korea; dbals28@inu.ac.kr

**Keywords:** transportation, mobile robots, smart buildings, crowd, surveillance

## Abstract

Recent transportation systems are operated by cooperative factors including mobile robots, smart vehicles, and intelligent management. It is highly anticipated that the surveillance using mobile robots can be utilized in complex transportation areas where the high accuracy is required. In this paper, we introduce a crowd surveillance system using mobile robots and intelligent vehicles to provide obstacle avoidance in transportation stations with a consideration of different moving strategies of the robots in an existing 2D area supported by line-based barriers and surveillance formations. Then, we formally define a problem that aims to minimize the distance traveled by a mobile robot, while also considering the speed of the mobile robot and avoiding the risk of collisions when the mobile robot moves to specific locations to fulfill crowd surveillance. To solve this problem, we propose two different schemes to provide improved surveillance that can be used even when considering speed. After that, various ideas are gathered to define conditions, set various settings, and modify them to evaluate their performances.

## 1. Introduction

Recently, transportation systems are operated with a combination of 5G, 6G, IoT (Internet of Things), IIoT (Industrial Internet of Things), Open RAN (Radio Access Network) and cooperative components such as mobile robots, smart vehicles, intelligence for the purpose of autonomous control, intelligent service, etc. [1,2,3,4,5,6,7,8,9,10,11,12,13]. It is highly expected that the affective surveillance based on virtual emotion detection is expanding its applicability to various industrial and academic areas to achieve various missions and tasks including terror prevention, patrol service, virtual emotion-based service, criminal tracking, maritime transportation monitoring, smart complex area surveillance, etc. [14,15,16,17].

Mobile robots are increasingly being used in various fields with 5G, 6G, IoT, IIoT and the scope of their use is expanding to various environments such as industries, logistics, factories, and hospitals [18,19,20,21,22,23,24]. Mobile robots have various advantages, providing the ability to move in small spaces and perform tasks efficiently in complex and obstacle-filled environments. In addition, mobile robots are equipped with functions that can detect the surrounding environment, identify locations, plan routes, and control movement. This allows you to move along the desired path and perform your work. Taking advantage of these advantages, mobile robots can be used for various purposes even in places such as transportation areas, theme parks. In particular, it is anticipated that mobile robots and relevant detection technologies can take critical role of surveillance and patrol area [25,26,27,28,29,30,31,32]. In [33], the efficient coverage with re-assignment strategy was studied for multi-robot long-term surveillance. The cooperative dual-task path planning scheme was studied for persistent surveillance supported by a group of unmanned ground vehicles [34]. Also, the automation kit for dual-mode military unmanned ground vehicle was introduced for the purpose of surveillance and security [35]. In [36], the novel robot navigation with deep reinforcement learning was proposed to consider crowd area. The robot motion planning scheme with obstacle avoidance was performed in [37]. The distributed multi-robot navigation system was investigated based on variational Bayesian model [38]. Moreover, a balanced task allocation and motion planning framework was developed to consider fuzzy time windows [39]. In [40], authors developed the multi-agent deep reinforcement learning (MARL) that considers the limited sensor capability, localization. Also, in [41], the distributed and autonomous event-driven cooperative scheme using multi-robots was proposed to monitor mobile objects.

Then, cooperative and intelligent mobile robot systems are needed to achieve joint goals by interacting with each other. To this end, efficient communication and cooperation methods between mobile robots must be established, and algorithms for effective teamwork must be designed. Technical problems such as collision avoidance, routing and task distribution also should be effectively addressed. Through these efforts, mobile robots can efficiently cooperate and complete complex tasks. Previous studies have dealt with mobile robot systems mainly in 2D environments, but lacked consideration for robot movement speed. As a result, collisions between robots were ignored and the resulting problems were not considered sufficiently. However, collisions between robots can occur in real-world environments, which can prevent some robots from performing tasks or follow unexpected travel paths.

In this study, we solve the problem of minimizing the total distance traveled by robots by dealing with how to perform monitoring tasks using mobile robots within square 2D zones. Initially, robots are randomly placed in the area, and then conditions are added to set the robots moving speed the same. The relocation path of the robot should be planned in advance by considering the speed of movement of the mobile robot. In this situation, obstacles present in a square area and the priority between them can be dealt with. Obstacles are randomly assigned priorities, which play an important role in determining our main behavior. Obstacles have their own priorities. This is randomly assigned, and high priority obstacles must be avoided before low priority obstacles. It is important to avoid high-priority obstacles, which should be avoided sequentially according to priority. However, if the priority of an obstacle is lower than or equal to a certain degree, the barrier is formed rather than avoided and the obstacle is ignored. In this paper, we provide the ability to build barriers in a given area using mobile robots.

Based on the above observations, the main contributions and contents of the paper can be summarized as follows.

- First, we introduce the crowd surveillance system using mobile robots and intelligent vehicles to provide obstacle avoidance in transportation stations and smart buildings.
- Then, we officially define a main research problem that aims to minimize the distance traveled by robots when the crowd surveillance is created in the given transportation area with smart buildings where there are obstacles and these obstacles are randomly assigned priorities of security levels and monitoring importance.
- To solve the problem by applying various settings and running multiple simulations, we develop two different schemes. One method is based on dividing a given area in half and separately making it as well as another method is to exclude coordinates by drawing lines overall.
- Moreover, we evaluate the performance of the proposed schemes based on numerical results by expansive simulations according to various settings and scenarios and provide discussions and analysis for the obtained outcomes.

The rest of the paper is organized as follows: Section 2 covers the system settings, assumptions, system conditions, problem definition and key terms. Section 3 represents the proposed schemes to resolve the defined problem. Then, in Section 4, the performances of the proposed algorithms are analyzed according to the earned numerical results through simulations. Section 5 concludes the paper.

## 2. System Overview and Problem Definition

In this section, we specify the system settings, assumptions, system conditions, the main research problem definition, and essential terms which are used in the proposed crowd surveillance system.

### 2.1. System Settings and Assumptions

The following settings and assumptions are applied to the proposed system.

- The given transportation station area is a 2D square area where there may exist obstacles. Each obstacle is given priority at random.
- Security importance in transportation station area is determined by the given priority and can be ignored if it is lower than a certain priority.
- The system components include mobile robots where each mobile robot has the equal speed of movement.
- Mobile robots have communication systems for efficient communication and cooperation.
- If a mobile robot crashes, it can not be used. The mobile robot moves the floor and moves to specific positions to provide crowd surveillance.
- Mobile robots can move freely in all areas.
- Mobile robots work together to determine the optimal path and placement to create crowd surveillance.

### 2.2. System Conditions

Mobile robots are used to support crowd surveillance that can cover the entire area with a given square area, which prevents collisions between mobile robots and minimizes the total travel distance of the robot. As a condition, first set the mobile robot’s moving speed to the same. By doing everything the same, the order and route must be adjusted when moving the mobile robot, which prevents conflicts and allows for cooperative work. It also reduces algorithm complexity by eliminating the need for ordering and routing. In addition, there are obstacles in the square area, so you should avoid them and barrier-rum them, but the obstacles have randomly set priorities. The higher this priority is, it means that it is an obstacle that should be avoided carefully, but if it is lower than a certain price, the formation of a barrier becomes more important than avoiding obstacles, and obstacles are ignored. Next, set a limit on the maximum travel distance of the robot. This limits the robot from moving more than a certain distance and helps to find the minimum travel distance. This limitation limits the range of movement of the robot, which can lead to efficient path selection. Finally, we need to set limits on the communication range of mobile robots. Robots must be within a certain distance to communicate or cooperate effectively with each other. This allows robots to exchange information, share current situations, and work together. These system conditions are used to control the elements required for a mobile robot system to efficiently generate barriers, prevent collisions and achieve minimum travel distances.

### 2.3. Problem Definition

We deal with the problem of efficiently providing crowd surveillance using mobile robots within a given square area. The core of this problem is to minimize the total distance traveled by robots while avoiding collisions between them. Here, there are obstacles within this zone, and these obstacles randomly take precedence. Crowd surveillance should be constructed while avoiding obstacles, but priorities are like indicating importance, and obstacles with priorities below a certain value may be ignored because they are less important than barrier formation. Components include mobile robots that move within square areas to form crowed surveillance. The robot’s moving speed must be the same, and the moving speed must be adjusted appropriately to prevent it from becoming too fast or slow. The robot’s communication range and maximum travel distance should also be restricted to minimize travel distance. Then, the key terms and main research problem for the proposed system are defined as follows.

**Definition 1** (Crowd transportation space)**.** *The crowd surveillance space, referred as CTS, is the transportation space to allow crowd environments and factors covering crowd pedestrians, vehicles, objects, signals to provide intelligent transportation services in regard to the large amount of information and data.*

**Definition 2** (Crowd surveillance)**.** *Let us suppose that there exists the targeted crowd transportation space, the group of mobile components with moving speed, the set of obstacles. The crowd surveillance, called as CrowdSurv, is to support the detection of the crowd groups including penetrations and requested objects in the given space.*

**Definition 3** (crowd surveillance total movement minimization problem)**.** *Given that a group of mobile robots, a set of obstacles in crowd surveillance space, the crowd surveillance total movement minimization, referred as CrowdSurvTMin, is to minimize a total distance traveled by mobile robots such that the crowd surveillance is provided with adjusted moving speed of robots and with avoiding obstacles as well as the required detection range and allowable maximum movement limit are satisfied in crowd surveillance space.*

Then, the objective function (1) of *CrowdSurvTMin* problem is to

(1) Minimizeμ

## 3. Proposed Schemes

In this section, two different algorithms are presented to reduce the total travel distance of the robot within a square zone and to obtain energy-efficient results with minimal total movements of mobile robots. Then, a description of the execution procedures for each algorithm is specified.

### 3.1. Algorithm 1: Half-Divided-Positioning

Now, we describe the first algorithm, called as, Algorithm 1: *Half-Divided-Positioning*. The execution stage of the algorithm is as follows:

- Check the given square area.
- Identify the location of randomly scattered mobile robots.
- Verify the location of obstacles in the area and identify randomly assigned priorities.
- Obstacles whose priority is below a certain value are excluded.
- Divide the area in half horizontally.
- A crowd surveillance is constituted by moving one robot at a time in two divided areas.
- Calculate the total distance traveled by mobile robots and return it as final output μ.

After dividing the entire area in half horizontally, the mobile robot in the ground is moved one by one from above and below at the same time to generate a crowd surveillance. At this time, the mobile robot moves to the nearest place it can go. This is also applied when avoiding obstacles, and the robot closest to them moves to avoid obstacles. If this happens, the robots will move in different areas. Otherwise, the collisions between the mobile robots will not occur.

**Figure 1:**
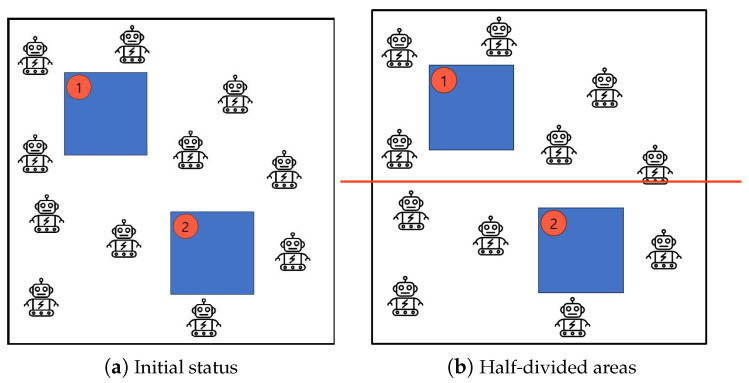
Initial status and the half-divided sub-regions by Algorithm 1: *Half-Divided-Positioning*.

Figure 1 depicts the initial status and the half-divided sub-regions by Algorithm 1: *Half-Divided-Positioning*. Figure 1a shows the initial state to cover a group of mobile robots and a set of obstacles with security priority. Also, Figure 1b represents the status of half-divided sub-regions by Algorithm 1: *Half-Divided-Positioning* so that the mobile robots determine the positioning or the moving strategy according the half-divided sub-regions.

**Figure 2:**
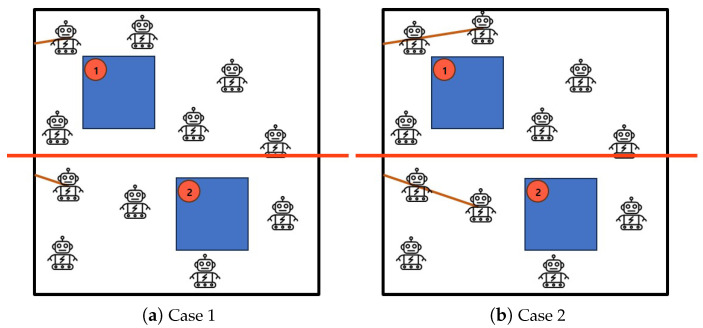

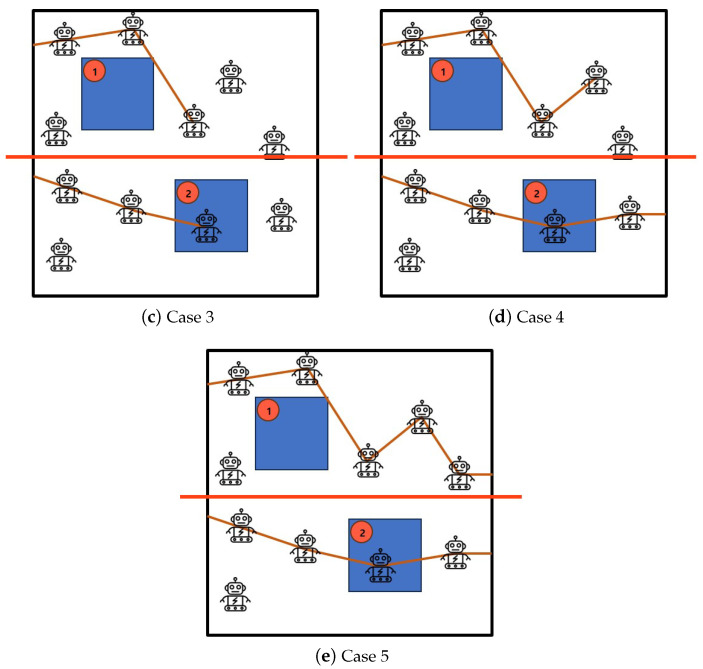
The execution cases of Algorithm 1: *Half-Divided-Positioning*.

Figure 2 shows implementation procedures and cases by Algorithm 1: *Half-Divided-Positioning*. Figure 2a describes the initial status with a verification of a group of mobile robots and a set of obstacles with priorities within the given area. Figure 2b depicts the first construction of crowd surveillance in the upper sub-region and lower sub-region, respectively. Figure 2c the case of obstacle avoidance with the highest priority in the upper sub-region while the crowd surveillance is maintained for obstacle the second priority in lower sub-region. Also, Figure 2d stands for the continuous construction of crowd surveillance in both the upper sub-region and lower sub-region. Figure 2e returns the completed result of crowd surveillance so that crowd surveillance are formed in both the upper sub-region and lower sub-region, respectively.
**Algorithm 1** ***Half-Divided-Positioning***Inputs: S,M,T, Output: μ
  1: accept the given area *S*;
  2: verify the positions of mobile robots *M* in *S*; 
  3: set M←∅;
  4: set total distance = 0;
  5: recognize the locations of obstacles *T* in *S*;
  6: assign the priority to each obstacle;
  7: divide *S* in half and set them as $S_{upper}$ and $S_{lower}$;
  8: **while** a crowd surveillance is not formed **do**
  9:       select a robot *m* in *M*;
10:       move *m* to min($S_{upper}$, $S_{lower}$);
11:       estimate the moving distance and add it to total distance;
12:       **if** the crowd surveillance is generated completely in *S* **then**
13:           exit
14: update total distance to μ;
15: return μ;

Moreover, the pseudocode of *Half-Divided-Positioning* is described in Algorithm 1 with clear representations.

### 3.2. Algorithm 2: Excluded-Coordinated-Movement

**Figure 3:**
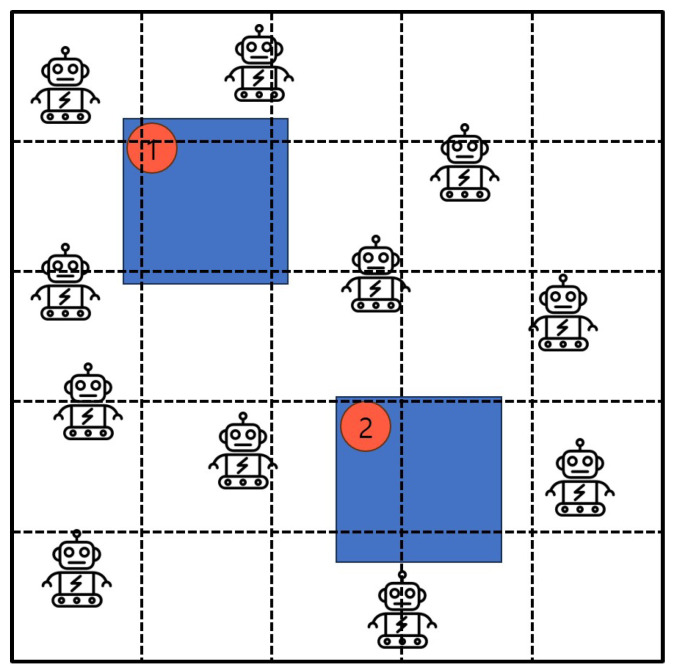
Initial status and the coordinated sub-areas according to virtual lines by Algorithm 2: *Excluded-Coordinated-Movement*.

Next, the second method, referred as Algorithm 2: *Excluded-Coordinated-Movement*, is presented to resolve the *CrowdSurvTMin* problem. The essential strategy of Algorithm 2: *Excluded-Coordinated-Movement* is to draw virtual lines to all given areas and to give coordinates to each other. Then, Algorithm 2: *Excluded-Coordinated-Movement* plans the movement path in advance using this coordinate, excluding the coordinates that passed bygone parts. Figure 3 presents the initial state which accepts transportation space with a group of mobile robots, a set of obstacles and its security priority toward securing crowd surveillance. As shown in Figure 3, the given transportation space is drawn by the dotted virtual lines so that the coordinated sub-regions are created. For the advantage of Algorithm 2: *Excluded-Coordinated-Movement,* the coordinates are used to make it easier to calculate the distance traveled, and the risk of collision is much lower because coordinates in the past are excluded. Also, since the route is planned in advance, it is efficient for avoiding obstacles.

Then, the execution procedures and steps of Algorithm 2: *Excluded-Coordinated-Movement* are explained as follows:

- Verify the given transportation space.
- Place mobile robots randomly in the given space.
- Identify the arbitrary location of mobile robots in all regions.
- Check the location of obstacles within the area and identify randomly assigned priorities.
- Exclude obstacles with priorities which do not exceed a certain value.
- Draw a line and give coordinates to all areas.
- Plan the robot’s travel path, excluding coordinates of past parts.
- Move the robot to a planned path and create a crowd surveillance.
- Estimate the total moving distance by mobile robots and return it as final output μ.

**Figure 4:**
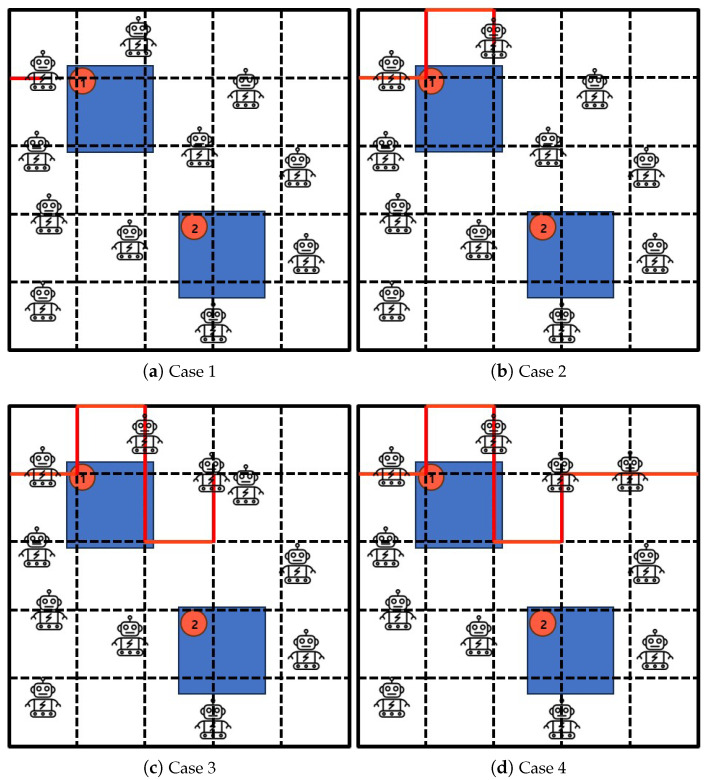
The implementation cases of Algorithm 2: *Excluded-Coordinated-Movement*.

Figure 4 shows execution procedures and cases by Algorithm 2: *Excluded-Coordinated-Movement*. Figure 4a presents the initial state covering a group of mobile robots and a set of obstacles with priorities within the given area where the coordinated sub-regions are drawn by virtual lines. Figure 4b depicts the scheduled status of mobile robot trajectory for crowd surveillance. Figure 4c represents the next planned status of mobile robots with the exclusion for the coordinates of past parts. Figure 4d stands for the completed result of crowd surveillance so that crowd surveillance is consequently generated with obstacle avoidance according to coordinated sub-regions.
**Algorithm 2** ***Excluded-Coordinated-Movement***
Inputs: S,M,T,b, Output: μ
  1: verify the given area *S*;
  2: locate a set of mobile robots *M* into *S*;
  3: check the positions of *M*;
  4: set M←∅;
  5: set total distance = 0;
  6: set a security priority bound *b*;
  7: identify the positions of obstacles *T* in *S*;
  8: **while** a priority is assigned to every obstacle *T* **do**
  9:       assign the priority *p* to an obstacle *t* of *T*;
10:       **if** *p* < *b* **then**
11:           exclude *t* from *T*;
12: generate the line-based coordinates in *S*;
13: **while** a crowd surveillance is not formed **do**
14:       select a robot *m* in *M*;
15:       move a robot to position for crowd surveillance with excluding coordinates of past parts;
16:       calculate the moving distance and add it to total distance;
17:       **if** the crowd surveillance is created completely in *S* **then**
18:           exit
19: update total distance to μ;
20: return μ;

In addition, the pseudocode of *Excluded-Coordinated-Movement* is presented in Algorithm 2 in more detail.

## 4. Experimental Evaluations and Discussions

### 4.1. Simulation Results

In this section, we analyze the outcomes of two different approaches: Algorithm 1: *Half-Divided-Positioning* and Algorithm 2: *Excluded-Coordinated-Movement* and their performances obtained from expansive simulations are demonstrated. For the simulation settings, the executed simulations have used the size of transportation space as 100 (width) by 100 (height) m, 150 by 150, 200 by 200, 250 by 250, respectively. The total number of mobile robots and vehicles *n* is between 100 and 250 and the communication range *r* is between 50 and 100 for each scenario graph. The required crowd surveillance level *l* is ranging from 4 to 7. Also, it is verified that every numerical result value of the total moving distance by mobile robots μ is the average value after 1000 cases with different settings and parameters with random locations of mobile robots and obstacles are performed. As a whole, our experiments for crowd surveillance with obstacle avoidance consist of three different groups of the applied scenarios according to the given space sizes, the total number of mobile robots, etc.

**Figure 5:**
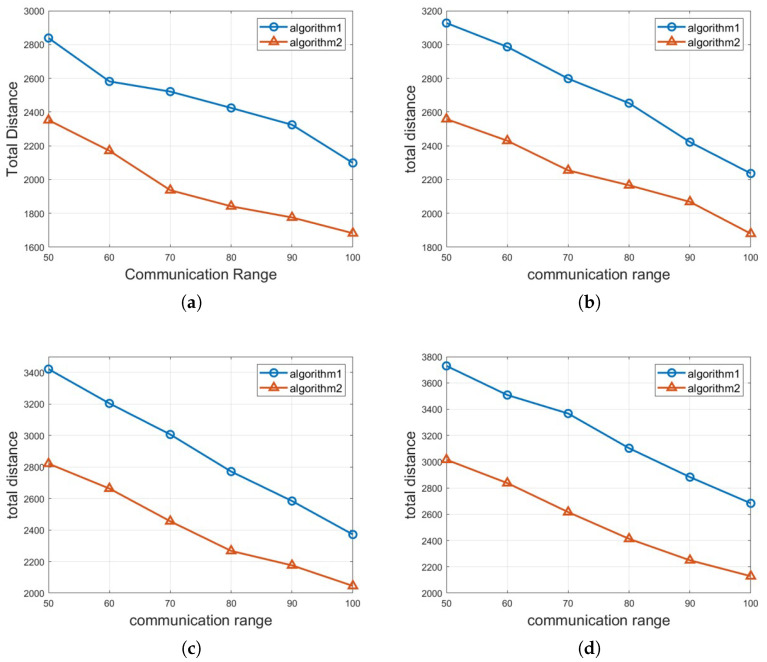
The total moving distance of μ by various number of mobile robots *n* in 100×100 crowd transportation space. (**a**) *n* = 100 in 100×100 crowd transportation space, (**b**) *n* = 150 in 100×100 crowd transportation space, (**c**) *n* = 200 in 100×100 crowd transportation space, (**d**) *n* = 250 in 100×100 crowd transportation space.

For the first group of experiment, we implement two different methods: Algorithm 1: *Half-Divided-Positioning* and Algorithm 2: *Excluded-Coordinated-Movement* with various number of mobile robots *n* in 100×100 crowd transportation space as shown in Figure 5. It is identified that the performance result graph represents two-axis so that X-coordinate stands for communication of mobile robot and Y-coordinate axis depicts the outcome of total distance μ. In the result graph, Algorithm 1: *Half-Divided-Positioning* is presented with circle marker with blue color, Algorithm 2: *Excluded-Coordinated-Movement* is shown with triangle marker with red color. Figure 5a,b demonstrate the results when *n* = 100 and *n* = 150 are put into the experiment. Figure 5c,d show the performance if *n* = 200 and *n* = 250 are set up with 100×100 crowd transportation space. As seen in Figure 5, we could check that the value of μ is decreasing as the communication range is increasing for both algorithms and Algorithm 2: *Excluded-Coordinated-Movement* has better performance than Algorithm 1: *Half-Divided-Positioning*.

**Figure 6:**
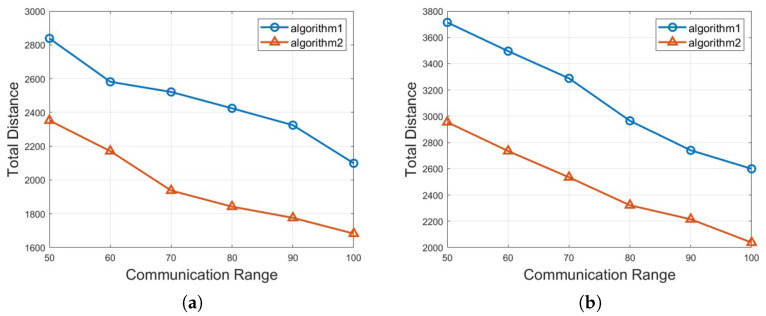

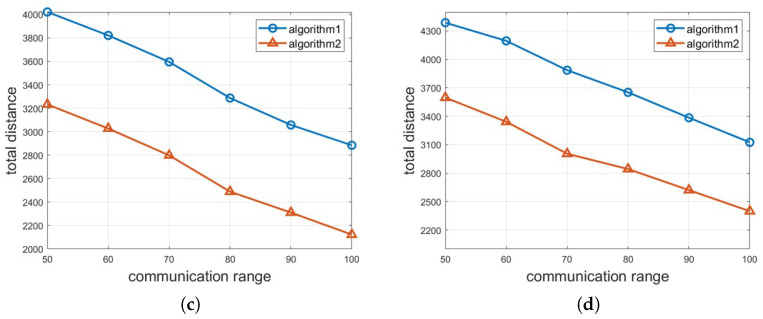
The total moving distance of μ by different size of crowd transportation space with the total number of mobile robots *n* = 100. (**a**) 100×100 crowd transportation space size with *n* = 100, (**b**) 150×150 crowd transportation space size with *n* = 100, (**c**) 200×200 crowd transportation space size with *n* = 100, (**d**) 250×250 crowd transportation space size with *n* = 100.

About the second group of simulation, we perform Algorithm 1: *Half-Divided-Positioning* and Algorithm 2: *Excluded-Coordinated-Movement* for a different size of crowd transportation space with the total number of mobile robots *n* = 100 as seen in Figure 6. Similar to the first group of experiment, Algorithm 1: *Half-Divided-Positioning* is presented with a circle marker with blue color and Algorithm 2: *Excluded-Coordinated-Movement* is shown with a triangle marker with red color on the result graph. Figure 6a,b show the performance if 100×100 and 150×150 size are considered as the crowd transportation space. Also, Figure 6c,d present the numerical result when 200×200 and 250×250 size are put into the given area size. According to Figure 6, we could verify that not only the total movement distance for mobile robots of μ decreases as the communication range increases but also Algorithm 2: *Excluded-Coordinated-Movement* makes a lower total moving distance than Algorithm 1: *Half-Divided-Positioning*.

**Figure 7:**
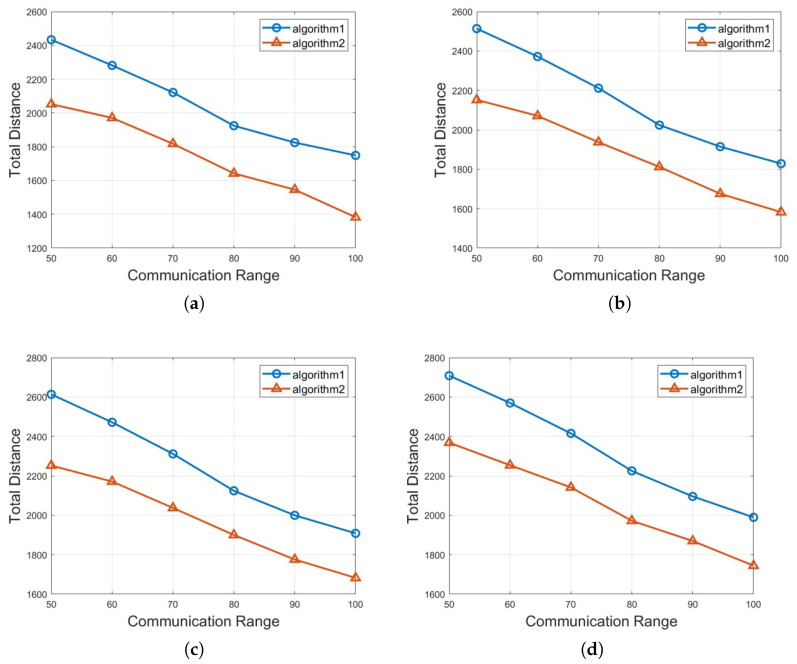
The total moving distance of μ by various crowd surveillance level *l* with *n* = 100 in 100×100 crowd transportation space. (**a**) crowd surveillance level *l* = 4 with *n* = 100 in 100×100 crowd transportation space, (**b**) crowd surveillance level *l* = 5 with *n* = 100 in 100×100 crowd transportation space, (**c**) crowd surveillance level *l* = 6 with *n* = 100 in 100×100 crowd transportation space, (**d**) crowd surveillance level *l* = 7 with *n* = 100 in 100×100 crowd transportation space.

For the third group of experiment, we execute Algorithm 1: *Half-Divided-Positioning* and Algorithm 2: *Excluded-Coordinated-Movement* by various crowd surveillance level *l* with *n* = 100 in 100×100 crowd transportation space as it is shown in Figure 7. It is noted that Algorithm 1: *Half-Divided-Positioning* is depicted with a circle marker with blue color and Algorithm 2: *Excluded-Coordinated-Movement* is represented with a triangle marker with red color on the result graph. Figure 7a,b demonstrate the performance when crowd surveillance level *l* = 4 and *l* = 5 are given, Figure 7c,d show the output value if crowd surveillance level *l* = 6 and *l* = 6 are requested, respectively. Based on Figure 7, we could check that the value of μ is decreasing as the communication range is increasing for both algorithms. Algorithm 2: *Excluded-Coordinated-Movement* returns the smaller moving distance μ than Algorithm 1: *Half-Divided-Positioning* for crowd surveillance. As a whole, it is confirmed that Algorithm 2: *Excluded-Coordinated-Movement* outperforms Algorithm 1: *Half-Divided-Positioning* consequently.

### 4.2. Complexity Analysis

When we calculate the complexity of the proposed Algorithm 1: *Half-Divided-Positioning* and Algorithm 2: *Excluded-Coordinated-Movement*, suppose that the given surveillance area is *S*, the total number of mobile robots is *n*, the total number of surveillance priority *k*, the total number of obstacles is *b* and the total number of coordinates *c*, where n>q, n>b, n>c.

First, if we deliberate on the complexity of Algorithm 1: *Half-Divided-Positioning*, it identifies the given surveillance area *S* within O(1) and it checks the location of randomly deployed mobile robots within O(n). Also, it verifies the assigned priorities within O(k) as well as checks the location of obstacles within O(b). Then, it divides the given area in half horizontally within O(1). Each mobile robot is positioned to two divided areas for avoiding obstacles *b* within b∗O(n). Then, the total distance of mobile robot is estimated within O(n) and return it as O(1). If so, the total number of iterations and calculations will be O(1)+O(n)+O(k)+O(1)+b∗O(n)+O(n)+O(1). Because *b* is constant, the asymptotic upper bound is O(n). Hence, the complexity of Algorithm 1: *Half-Divided-Positioning* is estimated as O(n).

Second, if we evaluate the complexity of Algorithm 2: *Excluded-Coordinated-Movement*, it verifies the requested surveillance area *S* within O(1) and places the mobile robots to the area *S* within O(n). It generates the arbitrary positions of mobile robots in all regions within O(n). It recognizes the location of obstacles within O(b) and the assigned priorities within O(k). Then, it draws the line and gives coordinates to each area within 2∗O(1)∗O(c). Also, it estimates the robot’s travel path excluding the previous parts within b∗O(n)∗O(c) for avoiding obstacles *b*. Then, the total distance of mobile robot is estimated within O(n) and return it as O(1). If so, the total number of iterations and calculations will be O(1)+O(n)+O(n)+O(b)+O(k)+2∗O(1)∗O(c)+b∗O(n)∗O(c)+O(1). Since *b* is constant, the asymptotic upper bound is O(n). Therefore, the complexity of Algorithm 2: *Excluded-Coordinated-Movement* is estimated as O(n).

## 5. Concluding Remarks

In this article, we introduced the crowd surveillance framework using mobile robots and intelligent vehicles to provide obstacle avoidance with security priorities in transportation stations, which deliberates on different moving strategies. Then, we made a formal definitions of key terms and the crowd surveillance total movement minimization problem. To find the solution of the defined problem, two different algorithms are devised to provide crowd surveillance completely in the give transportation space. Furthermore, the performance of the proposed schemes is analyzed based on numerical results by expansive simulations with various settings and scenarios.

## Data Availability

Data are contained within the article.

## Abbreviations

The following abbreviations are used in this manuscript:

IoT	Internet of Things
IIoT	Industrial Internet of Things
RAN	Radio Access Network
